# Review of Autism Profiles and Response to Sertraline in Fragile X Syndrome-Associated Autism vs. Non-syndromic Autism; Next Steps for Targeted Treatment

**DOI:** 10.3389/fneur.2020.581429

**Published:** 2020-10-30

**Authors:** Akash Rajaratnam, Laura Axelrod Potter, Hazel Maridith Barlahan Biag, Andrea Schneider, Ignacio Cortina Petrasic, Randi Jenssen Hagerman

**Affiliations:** ^1^MIND Institute, University of California, Davis, Sacramento, CA, United States; ^2^School of Medicine, Case Western Reserve University, Cleveland, OH, United States; ^3^School of Medicine, University of California, Davis, Sacramento, CA, United States; ^4^Department of Pediatrics, University of California Davis Health, Sacramento, CA, United States

**Keywords:** Fragile X Syndrome, Autism spectrum disorder (ASD), targeted treatment, sertraline, anxiety

## Abstract

Given significant genetic, molecular, and phenotypic overlaps, researchers have begun to investigate whether targeted treatments for Fragile X Syndrome (FXS) could also be beneficial for patients with Autism Spectrum Disorder (ASD). For example, low-dose sertraline, an SSRI, was used in two recent controlled trials in children with FXS and ASD. The first trial recruited 52 children with FXS, 32 of which were also diagnosed with ASD; the second trial recruited 58 children with non-syndromic ASD. One focus of the present study is to compare the response to sertraline between the FXS-associated ASD and non-syndromic ASD groups. Another focus is to compare baseline ASD-related characteristics between the groups and review these differences within the context of recent literature comparing these populations. Our comparison showed more severe ASD profiles in children with non-syndromic ASD vs. FXS-associated ASD. Regarding response to sertraline, the FXS-ASD group displayed significant improvements in language development, while the non-syndromic group did not show any significant improvements. One possible explanation for this differential response is the distinct anxiety profiles that are seen in these two groups. The heightened anxiety phenotype seen in those with FXS-ASD may have led to a greater relief of anxiety symptoms with sertraline compared to those with non-syndromic ASD; this, in turn, could have led to measurably greater developmental gains. Further research is required to solidify this connection between anxiety relief and developmental gains in these populations.

## Introduction

Autism spectrum disorder (ASD) is a behaviorally defined neurodevelopmental disorder that is characterized by impaired social and language development, repetitive behaviors, and restricted interests, as well as hyper or hypo-reactivity to sensory inputs. The etiology of ASD is complex and not well-defined, with over 500 different genetic mutations, as well as epigenetic interactions associated with the development of the disorder (1–3). Despite the often-multifactorial nature of the disorder, ASD can also be caused by single gene mutations. The most common single-gene cause of ASD is Fragile X Syndrome (FXS). Approximately 60% of males and 20% of females with FXS meet the criteria for ASD. The clinical presentation of FXS and idiopathic ASD overlap significantly, with a number of neurologic and behavioral characteristics seen in both conditions. These include language deficits, poor eye contact, repetitive behaviors, perseverative speech, hypersensitivity to environmental stimuli, ADHD, anxiety, and social deficits (4–6).

Unlike ASD, the etiology of FXS—the most common inherited cause of intellectual disability—is well-defined. It arises from a full mutation repeat expansion (>200 CGG repeats in the 5′ untranslated region) in the *FMR1* gene on the X chromosome. This expansion results in the methylation and subsequent silencing of the *FMR1* gene, leading to drastically reduced levels of FMRP, the protein product of *FMR1*. The amount of FMRP produced in an individual directly correlates with his or her degree of cognitive impairment, with higher levels found in affected individuals with IQ scores above 70 (7, 8). FMRP is an mRNA binding protein involved in the transport and translational regulation of a number of dendritic mRNAs (9). Acting at the ribosomal level, it regulates the translation of the proteins involved in synaptic maturation and integrity. Compromised FMRP levels lead to abnormal dendritic spine density, abnormal synaptic plasticity, and immature, elongated dendritic spine morphology (10, 11). The cognitive and behavioral deficits seen in FXS are associated with the synaptic dysfunction that stems from the loss of FMRP.

The molecular abnormalities seen in individuals with FXS have many similarities with those seen in individuals with ASD. Investigation of ASD-related genes, mostly through analysis of copy number variants (CNVs) and point mutations associated with ASD, has implicated three broad domains impacted in ASD pathophysiology: synaptic function, neuronal signaling and development, and chromatin regulation (12–16). Importantly, two of these three domains—synaptic function and neuronal signaling and development—are compromised in FXS as well. Recent studies have shown that FMRP binds to up to 50% of all genes associated with ASD (1), and the CNVs in genes responsible for postsynaptic regulation of FMRP are also associated with ASD (17). These genetic overlaps provide a possible explanation for why synaptic dysfunction and altered neuronal signaling are present in both ASD and FXS, as well as why the two conditions share such overlapping phenotypes.

Given these commonalities, researchers have started investigating whether treatments targeting the neurobiological dysfunction in FXS may also be effective in ASD. For example, two controlled trials of the selective serotonin reuptake inhibitor (SSRI) sertraline were recently completed, first in children with FXS and then in children with idiopathic (non-syndromic) ASD (18, 19). In young children with ASD, serotonin production and levels are abnormally low, especially in the first 5 years of life (20). This early developmental window is when synaptic formation occurs most rapidly. Thus, an SSRI could exert its greatest beneficial effect in these early years (18). In addition to improving low serotonin levels, there is evidence that SSRIs stimulate brain-derived neurotrophic factor (BDNF) in certain mouse models (21). BDNF is active at synapses and is involved in synaptic maturation, neurogenesis, and plasticity (21–23). Given the molecular deficits common to FXS and ASD, it is apparent why SSRIs have emerged as an intriguing option as a targeted treatment for both disorders.

The initial rationale for the two aforementioned trials came when a retrospective chart review showed that low-dose sertraline in 45 children with FXS, improved both expressive and receptive language trajectory (24). The first of the two controlled trials under comparison in this study enrolled 52 children with FXS from 2012 to 2015, 32 of these subjects were also diagnosed with ASD. The second controlled trial, which ran from 2015 to 2018, enrolled 58 children with non-syndromic ASD. One of the primary goals of this review is to compare the response to sertraline in the 32 children with fragile X syndrome-associated autism (FXS-ASD), as reported by Greiss Hess et al. (18), with the 58 children with non-syndromic ASD, as reported by Potter et al. (19). This review will also compare baseline data from the FXS-ASD and non-syndromic ASD groups concerning language development, autism severity, and overall cognition. These baseline comparisons will serve to strengthen the evidence cited in recent literature, which outlines that there are qualitatively different autism profiles in these two groups (6, 25). Understanding these differences, moreover, can have important implications for how we utilize targeted treatments in the future.

## Materials and Methods

### Participants and Design

Both trials under analysis were double-blind, placebo-controlled studies in children between the ages of 2 and 6 years. The two protocols were designed to be as similar as possible to maximize our ability to directly compare and contrast the effects on the two different populations. Both trials followed the same structure with a total of three study visits: an initial visit that included a detailed physical exam, diagnostic testing, and developmental testing; a 3-months visit that involved side effects and safety monitoring; and a 6-months visit during which testing was repeated to assess for developmental gains.

For the first trial, the inclusion criteria were molecular documentation of FXS, having a primary caregiver who was English speaking, and willingness to travel and participate in the trial. The exclusion criteria included whether they had a central nervous system (CNS) disease other than FXS or any other serious comorbid medical disorder. For the second trial, inclusion criteria were documentation of ASD as verified using both the Diagnostic and Statistical Manual of Mental Disorders, Fifth Edition (DSM-5) and Autism Diagnostic Observation Schedule, Second Edition (ADOS-2), concurrent enrollment in at least one community or school intervention for ASD, having a primary caregiver who was English speaking, and willingness to travel and participate in the trial. The exclusion criteria included a previous diagnosis of the FXS full mutation or identification of the full mutation on initial visit blood testing, current or past SSRI treatment, and any other serious comorbid medical disorders. Though individuals with FXS were excluded from this latter trial, there was one patient from this study whose autism was associated with another genetic syndrome—Beckwith-Wiedemann Syndrome. However, the other 57 children from this trial had purely idiopathic ASD not associated with a genetic syndrome.

The study drug was administered in liquid form (20 mg per mL). Subjects ages 2–3 years received sertraline liquid or placebo liquid in a dose of 2.5 mg per day (0.125 mL). Subjects ages 4–6 years received a dose of 5.0 mg per day (0.25 mL). The doses were based on those used in the retrospective study that originally suggested sertraline may help improve the trajectory of language development (24).

### Assessments

Developmental assessments for both studies were conducted in the clinic at the initial study visit and the final 6-months visit. Both trials included the following study assessments: Mullen Scales of Early Learning (MSEL), Clinical Global Impression—Improvement Scale (CGI-I), Preschool Language Scale—Fifth Edition (PLS-5), Sensory Processing Measure—Preschool Edition (SPM-P), and Vineland Adaptive Behavior Scale-II (VABS-II). Both trials conducted the ADOS-2 for diagnostic purposes at the initial study visit only. Despite the majority of assessments overlapping between both studies, some assessments were only used in one study. For example, the McArthur-Bates Communicative Development Inventories (CDI) and Parenting Stress Index—Fourth Edition (PSI-4) were only used in the FXS trial, while the Aberrant Behavior Checklist (ABC) was only used in the non-syndromic ASD trial. Only assessments used in both trials were analyzed in this comparison.

For both trials, language development as measured by the MSEL Expressive Language scores were the primary outcome measure. Accordingly, language development was a focus of our baseline comparison, which analyzes the baseline MSEL Expressive Language (EL) and Receptive Language (RL) raw scores, as well as PLS-5 Expressive Communication (EC) and Auditory Comprehension (AC) raw scores. The MSEL Early Learning Composite (ELC) standard score, thought to be representative of an IQ equivalent in children with ASD, will also be analyzed. Lastly, this comparison will use ADOS-2 Social Affect (SA) and Restrictive and Repetitive Behavior (RRB) scores to better characterize the distinct autism profiles seen in the two populations.

### Statistical Analysis

The baseline ADOS-2 scores, MSEL scores, and PLS-5 scores between the FXS-ASD and non-syndromic ASD groups were analyzed using non-parametric, two-sample Mann-Whitney *U*-tests carried out with the statistical package SPSS, version 26. Analysis of the response to sertraline with respect to MSEL EL and RL raw scores was carried out and described by Greiss Hess et al. and Potter et al., respectively (18, 19).

## Results

### Baseline Data

Our results showed that ADOS-2 SA scores (14.051 vs. 11.125; *p* < 0.01) as well as RRB scores (5.258 vs. 3.875; *p* < 0.005) were both significantly increased in the non-syndromic ASD group relative to the FXS-ASD group, indicating more severe autistic behaviors in the non-syndromic ASD group (Table 1). Effect sizes for SA and RRB scores showed small to medium strengths of association (*r* = 0.28 and *r* = 0.32, respectively). No differences in baseline MSEL EL (18.862 vs. 17.645; *p* = 0.769) or RL (22.344 vs. 21.806; *p* = 0.880) scores were found between the two groups, nor was there any difference in EC scores on PLS-5 (22.95 vs. 23.44; *p* = 0.61). However, the lower AC scores on PLS-5 in the non-syndromic ASD group relative to the FXS-ASD group approached statistical significance (22.70 vs. 27.19; *p* = 0.075) and had a small strength of association (*r* = 0.19), suggesting marginally more advanced auditory comprehension in those with FXS-ASD. Notably, the higher Early Learning Composite (ELC) standard score in the non-syndromic ASD group approached statistical significance as well (57.67 vs. 51.06; *p* = 0.077; *r* = 0.19).

**Table 1:** Comparison of baseline ADOS-2, MSEL, and PLS-5 baseline scores.

	**Non-syndromic ASD**			**Fragile X-associated ASD**		
	***N***	**Mean**	**Standard deviation**	***N***	**Mean**	**Standard deviation**
ADOS-2 SA score	58	13.98	4.54	32	11.13	4.44
ADOS-2 RRB score	58	5.26	2.08	32	3.88	1.58
MSEL EL raw score	58	18.96	10.92	31	17.65	8.65
MSEL RL raw score	58	22.34	9.78	31	22.10	7.63
MSEL ELC standard score	58	57.67	12.77	32	51.06	3.92
PLS-5 AC raw score	56	22.70	12.83	31	27.19	10.19
PLS-5 EC raw score	56	22.95	11.82	31	23.44	8.85
	**Mean difference**					**Effect size (*****r***)^*^
ADOS-2 SA score	2.85 (*p = 0.008)*					0.28
ADOS-2 RRB score	1.38 (*p = 0.002*)					0.32
MSEL EL raw score	1.31 (*p = 0.77)*					0.03
MSEL RL raw score	0.24 (*p = 0.88*)					0.02
MSEL ELC standard score	5.61 (*p = 0.077)*					0.19
PLS-5 AC raw score	4.49 (*p = 0.075)*					0.19
PLS-5 EC raw score	0.49 (*p = 0.61)*					0.05

* *Interpretation of Pearson r coefficient according to Cohen (1988): 0.1–0.3—small strength of association; 0.3–0.5—medium strength of association; 0.5–1.0—large strength of association (26)*.

^**^SA, Social Affect; RRB, Restrictive and Repetitive Behaviors; EL, Expressive Language; RL, Receptive Language; ELC, Early Learning Composite; AC, Auditory Comprehension; EC, Expressive Communication.

### Response to Sertraline

According to the MSEL EL raw scores, those with FXS-ASD experienced a significant improvement with sertraline vs. placebo (23.5 vs. 17.6; *p* < 0.005) (18), while those with non-syndromic ASD experienced no difference between sertraline treatment and placebo (20.3 vs. 21.8; *p* = 0.547) (19). Regarding the MSEL RL scores, there was no difference between sertraline and placebo in either the FXS-ASD group or the non-syndromic ASD group (18, 19).

## Discussion

One of the main goals of this study was to further contribute to the body of knowledge regarding phenotypic similarities and the differences between non-syndromic ASD and fragile X-associated ASD, and our results support the findings of recent studies showing qualitatively different autism profiles between these two populations. Wolff et al. (25), comparing age-matched boys with FXS-ASD and non-syndromic ASD, analyzed five ADOS-2 measures and found that boys with FXS-ASD were less impaired in regards to social smiling, quality of social overtures, and facial expressions relative to boys with non-syndromic ASD. Moreover, McDuffie et al. (6) found that boys with FXS-ASD demonstrated more social smiling, more motivation to engage in triadic interactions, and more non-verbal gestures relative to age-matched boys with non-syndromic ASD. Furthermore, among verbal participants, those with FXS-ASD were more likely to engage in social conversation. These reports of less social impairment in individuals with comorbid FXS and ASD are concordant with the results of the present study, which found significantly lower social affect domain scores in children with FXS-ASD relative to non-syndromic ASD.

These findings also demonstrate fewer restrictive and repetitive behaviors in children with FXS-ASD, which also supports McDuffie et al.' report that boys with FXS-ASD exhibit less insistence on sameness relative to boys with non-syndromic ASD (6). Regarding baseline language development, the FXS-ASD group showed modestly advanced auditory comprehension relative to the non-syndromic ASD group but no difference in expressive language or communication. Notably, MSEL ELC scores—thought to be an IQ equivalent for individuals with ASD (27)—were slightly higher in the non-syndromic ASD group. These results are again consistent with previous evidence showing higher levels of non-verbal IQ in non-syndromic ASD relative to FXS-ASD (6).

Understanding the differences and similarities between FXS-ASD and non-syndromic ASD profiles is important clinically, specifically in answering the question of whether treatments targeting the core symptoms of FXS can also be useful for individuals with non-syndromic ASD. Some speculate that the potential for common treatments for these two conditions negatively correlates with an increasing number of differences identified between the two conditions (6). This is a valid concern, and it may provide a rationale for why, contrary to what the authors hypothesized, sertraline did not stimulate language gains in children with non-syndromic ASD like it did in children with FXS-ASD.

We believe that the underlying basis for this incongruent response to sertraline can in part be explained by the differing anxiety profiles between the two groups. Individuals with FXS have more significant GABA deficits, leading to impaired habituation to sensory stimuli, greater sympathetic responses, and a heightened anxiety phenotype relative to individuals with non-syndromic ASD (19, 28). Recent studies have shown that certain components of ASD symptomatology seen in those with FXS-ASD, including repetition of words or phrases as well as gaze avoidance, may be due to heightened anxiety, secondary to FXS rather than solely due to the social impairment seen in those with ASD without FXS (29–31). This suggests different mechanistic underpinnings behind overlapping phenotypes between FXS-associated and non-syndromic ASD. Given this, it is possible that the FXS-ASD population, with a greater degree of anxiety than those with non-syndromic ASD, experienced more appreciable anxiety relief while taking sertraline. This, in turn, could have facilitated the behavioral or attentional improvements that led to measurably greater developmental and language gains by the end of the study (19, 30). Moreover, this anxiety link explains why, in our baseline analysis, the FXS-ASD group had better auditory comprehension than the non-syndromic ASD group despite equivalent levels of expressive communication. This supports the notion that children with FXS-ASD have a better understanding of language and communication than children with non-syndromic ASD at baseline, but their underlying anxiety manifests as an apparent deficit in expressive language.

These hypotheses must be viewed with caution, however, and have two main limitations. First, baseline levels of anxiety were not included in the study protocol described by Greiss Hess et al., so analysis of anxiety levels between the two study populations was not possible. Second, there is limited evidence that this low dose of sertraline is effective in treating anxiety, as the usual lowest starting dose for sertraline when treating pediatric anxiety and depression is 12.5–25 mg (32). However, these recommendations are typically for older children, as SSRIs are not frequently started in children under 6 years old and thus have not been well-studied in this age group. Furthermore, we have seen anecdotal evidence in our clinical practice that 2.5–5.0 mg of sertraline can improve anxiety in this very young patient population.

The next steps in regards to sertraline as a targeted treatment should involve narrowing focus on the study population in whom we have already seen demonstrable benefits, young children with FXS-ASD. Future research on the topic should involve clear pre and post-treatment anxiety levels to answer the question of whether this dosage of sertraline can improve anxiety in affected children of this age. It is important to note, though, that the complex and varying clinical picture seen in ASD can complicate the evaluation of anxiety in these patients. Thus, future studies that analyze this must be sure to evaluate the different possible manifestations of anxiety in these patients in addition to social withdrawal. This includes irritability—which can manifest itself in a number of ways such as tantrums, aggression, and self-injurious behavior—as well as hyperactivity behaviors such as excessive movement or inability to sit still. Thus, a detailed evaluation of anxiety in this patient population is required.

If this data is gathered and analyzed within the context of pre and post-treatment language development markers as well as ADOS-2 scores, it could elucidate whether improving anxiety will drive improvements in language development and autism severity, as the authors of this study hypothesize. Such data would accomplish three main goals: it would provide novel evidence that low-dose sertraline is effective in treating anxiety in this specific patient population. It would further solidify the argument that increased anxiety in FXS-ASD is the primary driver of social withdrawal, in contrast to the intrinsic social deficits seen in non-syndromic ASD. Finally, it would help optimize our use of targeted treatments within these populations in the future.

## Author Contributions

AR, LP, and RH contributed to the conception and design of the study. AR and HB performed the statistical analyses. AR and IP wrote the first draft of the manuscript. AS administered developmental and language assessments. All authors contributed to manuscript writing, revision, read, and approved the submitted version.

## Conflict of Interest

RH has received funding from Zynerba, Ovid, and the Azrieli Foundation for carrying out treatment studies in patients with fragile X syndrome. She has also consulted with Fulcrum, Ovid, and Zynerba regarding treatment studies in individuals with fragile X syndrome. The remaining authors declare that the research was conducted in the absence of any commercial or financial relationships that could be construed as a potential conflict of interest.
